# Simple and Safe Synthesis of Yolk-Shell-Structured Silicon/Carbon Composites with Enhanced Electrochemical Properties

**DOI:** 10.3390/molecules29061301

**Published:** 2024-03-14

**Authors:** Jinhuan Li, Min Wu, Quan Du, Gangpeng Zhai, Haiyong He

**Affiliations:** 1 Ningbo Institute of Materials Technology and Engineering, Chinese Academy of Sciences, Ningbo 315201, China; lijinhuan@nimte.ac.cn (J.L.); wumin@nimte.ac.cn (M.W.); duquan@nimte.ac.cn (Q.D.); zhaigangpeng@nimte.ac.cn (G.Z.); 2 University of Chinese Academy of Sciences, Beijing 101400, China

**Keywords:** Si anode, yolk-shell structure, lithium-ion battery, annealing process

## Abstract

With its substantial theoretical capacity, silicon (Si) is a prospective anode material for high-energy-density lithium-ion batteries (LIBs). However, the challenges of a substantial volume expansion and inferior conductivity in Si-based anodes restrict the electrochemical stability. To address this, a yolk-shell-structured Si–carbon composite, featuring adjustable void sizes, was synthesized using tin (Sn) as a template. A uniform coating of tin oxide (SnO_2_) on the surface of nano-Si particles was achieved through a simple annealing process. This approach enables the removal of the template with concentrated hydrochloric acid (HCl) instead of hydrofluoric acid (HF), thereby reducing toxicity and corrosiveness. The conductivity of Si@void@Carbon (Si@void@C) was further enhanced by using a high-conductivity carbon layer derived from pitch. By incorporating an internal void, this yolk-shell structure effectively enhanced the low Li^+^/electron conductivity and accommodated the large volume change of Si. Si@void@C demonstrated an excellent electrochemical performance, retaining a discharge capacity of 735.3 mAh g^−1^ after 100 cycles at 1.0 A g^−1^. Even at a high current density of 2.0 A g^−1^, Si@void@C still maintained a discharge capacity of 1238.5 mAh g^−1^.

## 1. Introduction

As innovative green energy storage devices, LIBs have found extensive applications across various energy storage fields [1,2]. With the swift advancements in electric vehicles and consumer electronics in recent years, the demand for LIBs with higher energy densities has escalated [3,4,5]. Owing to its high theoretical capacity of 4200 mAh g^−1^ and abundant reserves, Si is regarded as the most promising high-energy anode material [6,7]. However, Si anodes undergo a nearly 300% volume change [8,9] caused by the massive insertion/extraction of lithium during the cycle. These significant strains disrupt the conductive network between active materials, leading to electrode material fracture or morphological changes in the electrode. Ultimately, these electrode materials fall off from the current collector, losing electrical contact and causing a rapid decline in the reversible capacity of the electrode [10]. In addition, the semiconductor properties of Si lead to poor conductivity, which compromises the rate performance of Si anode [11,12].

Numerous strategies have been put forward to tackle these challenges. One effective approach to improve the cycling stability of Si-based anodes is to utilize Si nanostructures, such as nanoparticles [13], nanowires [14], and nanotubes [15]. The nanoscale dimensions can mitigate overall stress and shorten the path of charge transfer [16,17]. Liu et al. [18] found that particles with diameters less than 150 nm are less prone to cracking during the lithiation and delithiation processes. However, this strategy falls short for industrial applications due to their poor conductivity. To enhance the poor electronic conductivity of Si, the application of conductive coatings or conductive matrix materials has been proposed [19,20]. Coatings of carbon, metals, or conductive polymers can establish a conductive pathway, facilitating electron transfer and enhancing rate performance [21,22,23]. Lee et al. [24] used electrospinning to synthesize a well-layered Si-Sn-C composite with multi-layered structures using SnO_2_ as precursors for Sn nanoparticles. Through high-temperature carbothermal reduction, SnO_2_ nanoparticles were transformed into metallic Sn nanoparticles, preventing Sn agglomeration during the heat treatment process, and it resulted in a Si-Sn-C composite with an excellent rate performance (720 mAh g^−1^ at 10,000 mA g^−1^). However, both Si and Sn undergo volume expansion due to alloying reactions with Li^+^ [25]. Cui et al. [26] introduced a yolk-shell structure that provides sufficient space for expansion and contraction during lithiation and delithiation, protecting the electrode from pulverization and retaining 74% capacity after 1000 cycles. However, the use of HF for etching the SiO_2_ template is highly toxic and presents environmental challenges, hindering its industrial application. A wealth of research demonstrates that the corrosive and toxic properties of HCl are significantly less than those of HF. Furthermore, the disposal of HCl waste is comparatively safer and simpler, and methods such as water absorption and alkali solution neutralization are commonly utilized [27].

This paper presents a straightforward and safe method for synthesizing a yolk-shell Si–carbon composite. The process began with the mixing of Si (Supplementary Figure S1) and anhydrous tin chloride (SnCl_2_). An annealing process [28] was then employed to coat SnO_2_ onto the surface of Si nanoparticles. The thickness of the void within the yolk-shell was adjusted by controlling the weight ratio between Si and SnCl_2_ (the samples with Si:SnCl_2_ ratios of 2:1, 2:3, and 2:5 were named Si@void@C-1, Si@void@C-2, and Si@void@C-3, respectively). Spray-drying [29] was used to form a spherical Si@SnO_2_ (Figure S2) composite. Carbon coating was achieved through the liquid-phase method [30], which allows for the uniform coating of pitch on the composite. In the final step, HCl was used instead of the toxic HF to remove the template, ensuring a safer experimental procedure. After filtration and washing, the yolk-shell-structured Si@void@C composite was obtained. The fabricated yolk-shell structure exhibited a superior electrochemical performance, with the Si@void@C electrode maintaining a discharge capacity of 735.3 mAh g^−1^ after 100 cycles at 1.0 A g^−1^. Even at a high current density of 2.0 A g^−1^, Si@void@C sustained a discharge capacity of 1238.5 mAh g^−1^.

## 2. Results and Discussion

**Figure 1:**
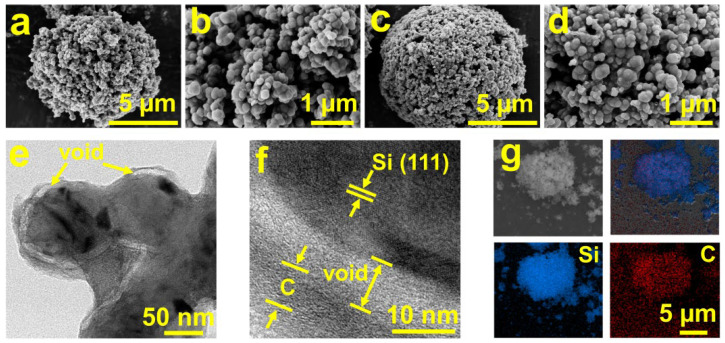
SEM of (**a**,**b**) Si@Sn@C, (**c**,**d**) Si@void@C. (**e**,**f**) TEM of Si@void@C. (**g**) Element mapping images (Si and C) of Si@void@C.

To alleviate the issue of volume expansion in Si anodes, a Si–carbon composite with internal voids were prepared. Scanning Electron Microscopy (SEM) images showed that both the Si@tin@carbon (Si@Sn@C, Figure 1a) and Si@void@C (Figure 1c) composites formed uniform spherical structures. The structures of Si@Sn@C and Si@void@C composites were similar after acid washing, indicating that etching with HCl has little impact on the overall morphology of the composites. Further magnified SEM images in Figure 1b,d reveal that these micron-sized spherical particles are composed of smaller nanoparticles. These nanoparticles are interspersed with numerous irregularly distributed pores, facilitating the infiltration of the electrolyte into the electrode material. The Transmission Electron Microscopy (TEM) image in Figure 1e shows a clear void between the Si nanoparticles and the carbon coating layer, indicating the successful preparation of a yolk-shell-structured Si–carbon composite. Figure 1f illustrates that the (111) crystal plane of polycrystalline Si [31] is present inside the spherical structure, which is coated externally with a high-conductivity amorphous carbon layer. And there is a 7–9 nm void between the external carbon layer and the internal Si (Figure S3). The average particle size of the selected Si is between 80 and 100 nm, which is below the critical rupture value of 150 nm for Si. After being coated with carbon and etched by HCl, the particle size of the obtained Si@void@C increased to 100–120 nm. Additionally, the calculated volume ratio of the Si yolk compared to the void space was roughly between 1:1.7 and 1:2.0. This suggests that the structure can effectively provide a buffering space for the volume expansion of Si, thus enhancing the durability and performance of the composite [32]. The EDS images of the Si@void@C composite (Figure 1g) show that Si and C elements are uniformly distributed on the spherical particles, suggesting the successful coating of the Si nanoparticles with pitch using the liquid-phase method. In contrast to the EDS image of Si@void@C, the Si@Sn@C also contains uniformly distributed Sn elements in addition to the Si and C elements (Figure S4).

**Figure 2:**
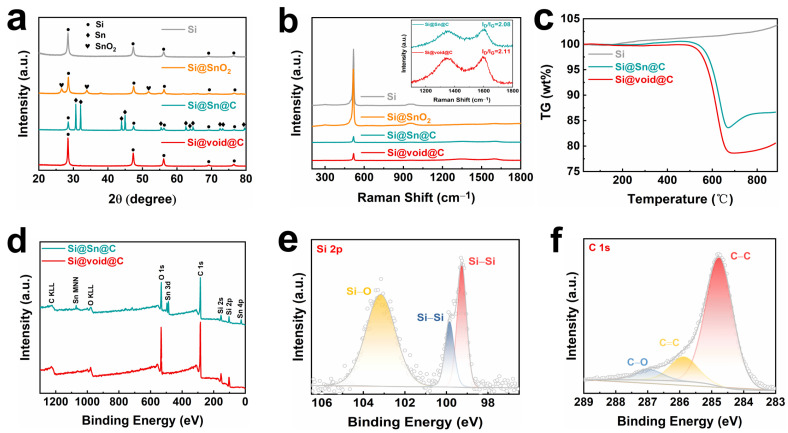
(**a**) XRD patterns. (**b**) Raman scattering spectra. (**c**) TG curves. XPS (**d**) full spectra and high−resolution spectra of Si@void@C: (**e**) Si 2p and (**f**) C 1s.

The phase composition of the composite was further studied through X-ray diffraction (XRD) patterns. As shown in Figure 2a, all composites exhibit diffraction peaks of crystalline Si at 2θ = 28.5°, 47.7°, 56.2°, 69.2°, and 76.3°, corresponding to the (111), (220), (311), (400), and (331) planes of Si [33], respectively. In the Si@SnO_2_ composite, characteristic peaks of SnO_2_ appear at 2θ = 26.5°, 33.8°, and 51.7°, corresponding to the (110), (101), and (211) planes of SnO_2_ [34], respectively, indicating that a layer of SnO_2_ is successfully coated on the surface of Si through the annealing process. For the Si@Sn@C composite, characteristic peaks of Sn appear at 2θ = 30.6°, 31.9°, 43.9°, 44.9°, 55.3°, 62.6°, 63.8°, 64.6°, 72.4°, 73.2°, and 79.5°, corresponding to the (200), (101), (220), (211), (101), (112), (400), (321), (420), (411), and (312) planes of Sn [35], respectively, further proving that SnO_2_ is reduced to Sn during the carbonization process. After etching the metal Sn with HCl, only the diffraction peaks of Si existed in the XRD pattern of the Si@void@C composite, indicating that the Sn had been completely removed, and it is feasible to prepare a yolk-shell-structured Si–carbon composite using Sn as a template on the surface.

As depicted in the Raman spectra (Figure 2b), a distinct peak emerges around 515 cm^−1^, aligning with the standard Raman peak of Si [36] crystals. The Raman spectra for the Si@Sn@C and Si@void@C composites exhibit two broad peaks at 1320 cm^−1^ and 1590 cm^−1^, which are attributable to the D band of sp^3^ disordered carbon and the G band of sp^2^ graphite [37,38], respectively. The ratio of the intensities of the D and G peaks (I_D_/I_G_) serves as a measure of the graphitization level of carbon materials. The I_D_/I_G_ values for Si@Sn@C and Si@void@C are 2.08 and 2.11, respectively.

Thermogravimetric analysis (TGA) was performed on the Si, Si@Sn@C, and Si@void@C composites, as shown in Figure 2c and Figure S5. During the process from 25 °C to 900 °C, Si reacted with oxygen to form an oxide, corresponding to a 3.67% mass increase. Within the temperature range of 25 °C to 550 °C, the main reaction for the Si@Sn@C and Si@void@C composites was the oxidation of Si and Sn; hence, a slight upward shift in the thermogravimetric (TG) curve was noted. After calculations, it was found that the oxidation of the carbon material led to weight losses of 17.4% and 19.7% for Si@Sn@C and Si@void@C composites from 550 °C to 700 °C, respectively. The higher weight reduction in Si@void@C compared to Si@Sn@C from 550 °C to 700 °C was due to its higher carbon content. The subsequent rise in the TG curve from 700 °C to 900 °C can be attributed to the further oxidation of Si and Sn.

The X-ray photoelectron spectroscopy (XPS) spectra of Si@Sn@C and Si@void@C composites are depicted in Figure 2d–f. The full spectra in Figure 2d confirm the coexistence of C, Si, Sn, and O elements in Si@Sn@C, while the fine spectra in Figure S6 further illustrate the presence of Sn 3d (Sn 3d_5/2_, Sn 3d_3/2_) [39]. In contrast, the absence of Sn in Si@void@C further verifies that the metal Sn was etched away by HCl, which is consistent with the XRD results. The high-resolution spectra in Figure 2e show two peaks at 99.6 eV and 103.3 eV, corresponding to the binding energies of Si-Si and Si-O [40], respectively. In Figure 2f, the high-resolution spectra of C 1s can be fitted into three peaks, corresponding to the sp^2^ C-C bond at 284.8 eV, the C=C bond at 286.04 eV, and the C-O bond at 287.14 eV [41].

**Figure 3:**
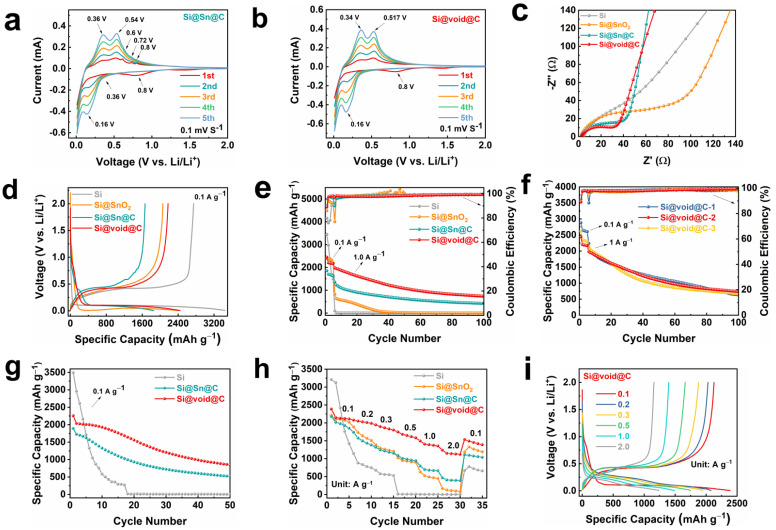
CV profile of (**a**) Si@Sn@C and (**b**) Si@void@C at 0.1 mV s^−1^. (**c**) Nyquist plots. (**d**) Charge/discharge profiles. (**e**,**f**) Cycling performance at 1 A g^−1^ and corresponding Coulombic efficiency. (**g**) Cycling performance at 0.1 A g^−1^. (**h**) Rate capability at current densities between 0.1 and 2 A g^−1^ and (**i**) corresponding charge/discharge profiles of Si@void@C.

Initially, the Si@Sn@C and Si@void@C electrodes were assembled into half-cells and subjected to cyclic voltammetry (CV) tests. Figure 3a,b display the first five cyclic voltammetry curves of the Si@Sn@C and Si@void@C electrodes, respectively, within a voltage range of 0.01–3.0 V (vs. Li/Li^+^) at a scan rate of 0.1 mV s^−1^. At the first cycle, a broad reduction peak appears at 0.8 V in the CV curve of Si@Sn@C and Si@void@C, which is related to the formation of a solid electrolyte interface (SEI) film [42] on the electrode surface due to the chemical reaction between the electrode material and the electrolyte. The SEI film is a good electronic insulator and a conductor of Li^+^, which can freely intercalate and deintercalate through this passivation film. The disappearance of this reduction peak from the second cycle confirms that the SEI film formed in the first cycle is stable. The reduction peak near 0.16 V at Si@Sn@C and Si@void@C can be attributed to the formation of a Li-Si alloy by Li^+^ and Si nanoparticles. The oxidation peaks at 0.36/0.34 V and 0.54/0.517 V correspond to the dealloying of the Li-Si alloy [43] in Si@Sn@C/Si@void@C. In the initial few cycles, the intensity of the reduction and oxidation peaks gradually increases with the progress of the cycle, resulting from the gradual electrochemical activation processes of the electrode. Unlike the Si@void@C anode, a reduction peak can be observed at 0.36 V in the CV curve of Si@Sn@C, which can be attributed to the formation of a Li-Sn alloy. And three weak oxidation peaks exist at 0.6 V, 0.72 V, and 0.8 V [44], which can be attributed to the lithium extraction process of Sn (Figure S7). Therefore, the CV curve indicates that, in addition to the alloying reaction between Si and Li^+^ to form Li-Si alloy in Si@Sn@C, Sn can also undergo an alloying reaction with Li^+^ to form a Li-Sn alloy. Compared with the CV curve at the same scan rate, the Si@void@C electrode has a lower extraction potential and a higher response current than the Si@Sn@C electrode, indicating that Si@void@C enhances the Li^+^ transport kinetics.

Subsequently, the electrochemical impedance spectra (EIS) of all samples were collected to examine the influence of structure on electron/ion transport, as depicted in Figure 3c. Prior to cycling, a single semicircle is noticeable in the high-frequency region of the Nyquist plots, corresponding to the charge transfer resistance between the active materials and the electrolyte interface. In this high-frequency region, the semicircle diameter for the Si@void@C electrode is smaller than other composites. The yolk-shell structure effectively inhibits direct contact between nano-Si and the electrolyte, thus reducing contact resistance and promoting charge transfer [45]. The optimization of interface and kinetics throughout the cycling process benefits the fast and stable storage of Li^+^ within Si@void@C.

The electrochemical performance of Si and the Si@SnO_2_, Si@Sn@C, and Si@void@C composites was studied by assembling half-cells, as shown in Figure 3d–i. For all the studied anodes shown in Figure 3e,f, the initial five cycles were activated at a current density of 0.1 A g^−1^, and subsequent cycles were performed at 1 A g^−1^. Figure 3d presents the first charge–discharge curves of the composite, where a long plateau appears below 0.1 V in the first discharge curve, attributable to the lithiation process of crystalline Si [46]. From Figure 3d, it can also be discerned that the Si@void@C electrode exhibits less polarization than other anodes. Figure 3e shows the long-cycle curves of the composite, with the initial discharge capacities of the Si, Si@SnO_2_, Si@Sn@C, and Si@void@C electrodes being 3441.9, 2394.9, 1851.4, and 2442.1 mAh g^−1^, respectively, and their corresponding initial Coulombic efficiencies (ICE) being 79.8%, 85.9%, 89.9%, and 89.1%, respectively (Figure S8). In terms of cycle stability, the capacity of the Si anode plummets after five cycles, with only 95 mAh g^−1^ remaining by the sixth cycle. The capacity of Si@SnO_2_ gradually falls below 100 mAh g^−1^ after 30 cycles, which is an improvement in electrochemical performance compared to pure Si anodes. This is due to the SnO_2_ layer in the Si@SnO_2_ electrode undergoing a transformation to produce Li_2_O and a Li-Sn alloy during lithiation [46,47], which protects nano-Si particles from electrolyte corrosion and enhances conductivity. Therefore, coating the surface of Si with a layer of metal oxide can effectively enhance conductivity and cycle stability. By coating the surface of the composite with a carbon layer, the conductivity of the Si@Sn@C anode is improved, thereby further enhancing the cycle stability of the composite. After 100 cycles at 1.0 A g^−1^, the capacity of the Si@Sn@C electrode still remains at 422.6 mAh g^−1^. However, the capacity of the Si@void@C electrode remains at 735.3 mAh g^−1^ after 100 cycles at 1.0 A g^−1^, which is 312.7 mAh g^−1^ higher than that of the Si@Sn@C electrode. This is due to the unique yolk-shell structure of the Si@void@C anode, which reserves space for Si and effectively mitigates the volume expansion of Si. The external carbon layer not only enhances the conductivity of the composite, but also serves as a mechanical support to prevent the pulverization and fracture of Si particles. Figure 3f presents the long-term cycling performance of the yolk-shell-structured Si–carbon composite with different void sizes. As can be seen from the figure, the initial discharge capacities of Si@void@C-1, Si@void@C-2, and Si@void@C-3 are 2881, 2442, and 2618 mAh g^−1^, respectively. After 100 cycles, the capacities decrease to 629, 735, and 664 mAh g^−1^, respectively. Hence, the composite exhibits a more stable electrochemical performance when the weight ratio of Si to SnCl_2_ is 2:3. Figure 3g presents the long-cycle chart of the composite at 0.1 A g^−1^, with the discharge capacity of the Si@void@C anode remaining at 849 mAh g^−1^ after 50 cycles, which is significantly higher than the capacities of Si (6 mAh g^−1^) and Si@Sn@C (522 mAh g^−1^).

In practical applications, rate performance is another important characteristic of electrode materials, as shown in Figure 3h,i and Figure S9. The reversible specific capacities of Si@void@C at current densities of 0.1, 0.2, 0.3, 0.5, and 1.0 A g^−1^ are 2387.8, 2078.0, 1944.8, 1748.0, and 1494.5 mAh g^−1^, respectively. In contrast, the discharge specific capacities of Si@Sn@C and Si@SnO_2_ at the same current densities are 2181.9/2215.5, 1736.6/1887.3, 1321.4/1385.8, 1088.9/1025.9, and 796.9/634.0 mAh g^−1^, respectively, which are significantly lower than Si@void@C. Even at a high current density of 2 A g^−1^, Si@void@C can still provide a discharge specific capacity of 1238.5 mAh g^−1^, which is 772.4 and 1067.4 mAh g^−1^ higher than Si@Sn@C (466.1 mAh g^−1^) and Si@SnO_2_ (171.1 mAh g^−1^), respectively. When the current density is reset to 0.1 A g^−1^, the discharge specific capacity of Si@void@C also recovers to 1533.8 mAh g^−1^, with a capacity retention rate of 64.2%. Therefore, the prepared Si@void@C has excellent rate performance, which is due to its internal voids providing a buffer space for the volume expansion of Si, thereby preventing the pulverization of Si. Furthermore, the conductivity of the composite is improved by the carbon layer derived from pitch within the yolk-shell structure.

**Figure 4:**
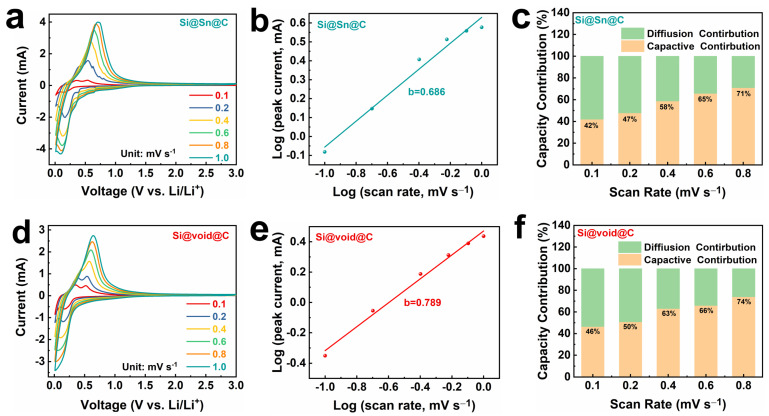
(**a**,**d**) CV curves of Si@Sn@C and Si@void@C electrodes at different scan rates from 0.1 to 1.0 mV s^−1^. (**b**,**e**) b-value analysis using the relationship between the peak currents and the scan rates. (**c**,**f**) Contribution ratio of capacitance response under different scanning rates.

To delve deeper into the electrochemical kinetics of lithium storage in Si@Sn@C and Si@void@C, the CV curves of the composite at scan rates between 0.1 and 1.0 mV s^−1^ are illustrated in Figure 4a,d. As can be seen from the CV curves, a slight shift in the oxidation peaks of the composite occurs with increasing scan rate, indicating a corresponding increase in the polarization of these materials. The kinetic behavior of an electrode can be determined using Equation (1):

(1) $$i=av^{b}$$

where i denotes current, ν signifies scan rate, and a and b are adjustable parameters [48]. A b value of 0.5 signifies diffusion-dominated redox-reaction kinetics, while a b value of 1.0 corresponds to surface-controlled redox-reaction kinetics. Figure 4b,e reveal b values of 0.686 and 0.789, respectively, implying a mixed electrochemical kinetics mechanism in the Si@Sn@C and Si@void@C electrodes. The contribution ratio of the reaction rates of the Si@Sn@C and Si@void@C electrodes can be computed using Formula (2):

(2) $$i=k_{1}v+k_{2}v^{0.5}$$

where $k_{1}v$ and $k_{2}v^{0.5}$ represent the diffusion-controlled and surface-controlled capacities [49,50], respectively. Figure 4c,f demonstrate that the capacitive contribution ratios of Si@Sn@C and Si@void@C at a scan rate of 0.8 mV s^−1^ are 71% and 74%, respectively. There is a notable 28% rise in the capacitive contribution ratios of the Si@void@C electrode, increasing from 46% at a scan rate of 0.1 mV s^−1^ to 74% at 0.8 mV s^−1^. With a higher capacitive contribution ratio, Si@void@C exhibits faster reaction kinetics. These results align with the excellent rate performance at varying current densities, suggesting that the yolk-shell structure aids in enhancing the electrochemical performance of Si@void@C.

**Figure 5:**
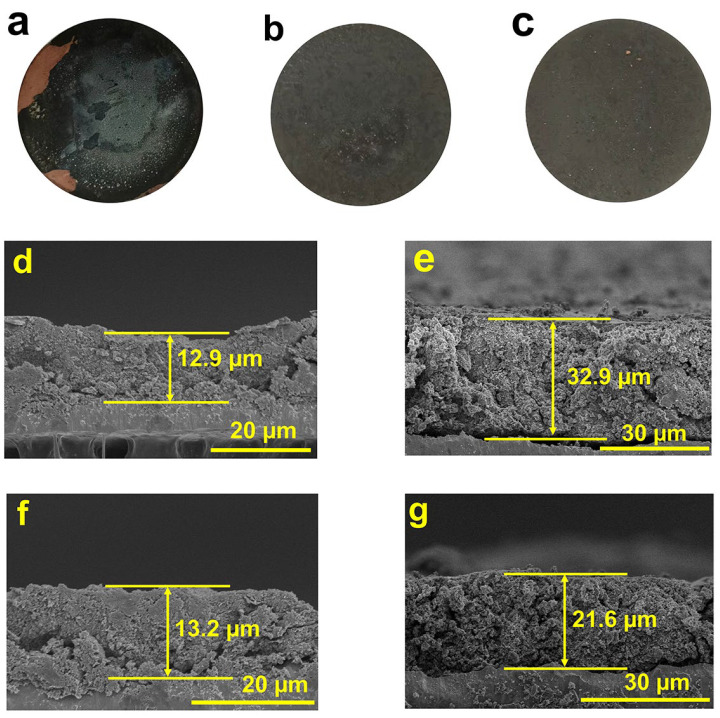
(**a**–**c**) Images of Si, Si@Sn@C, and Si@void@C electrodes after 100 cycles at 1 A g^−1^. Cross section SEM images of (**d**) Si@Sn@C and (**f**) Si@void@C before cycling, and (**e**) Si@Sn@C and (**g**) Si@void@C after 100 cycles at 1 A g^−1^.

To further investigate the characteristics of the materials, SEM cross-sectional images of Si@Sn@C and Si@void@C are observed before and after 100 cycles at 1A g^−1^. As observed in Figure 5a, due to the significant volume expansion of Si, the active material loses electrical contact, leading to a substantial detachment of the active material from the current collector after 100 cycles. However, the electrode of Si@Sn@C (Figure 5b) and Si@void@C (Figure 5c) remains intact. A further comparison of the cross-sections of the composite before and after cycling reveals that the thickness of the Si@Sn@C electrode increases from an initial 12.9 μm to 32.9 μm, with visible cracks appearing between the active materials. Given that both Si and Sn experience different degrees of volume expansion during the alloying process, Si@Sn@C cannot effectively alleviate the problem of volume expansion. The thickness of the Si@void@C electrode increases from the initial 13.2 μm to 21.6 μm, which is 11.3 μm smaller than Si@Sn@C. The significantly smaller volume change in the Si@void@C electrode compared to the Si@Sn@C electrode can be attributed to the yolk-shell structure of the Si@void@C electrode, which provides internal space to accommodate the volume expansion of Si, and the external carbon layer, which not only serves as a mechanical support but also boosts conductivity, thereby preserving the structural integrity.

## 3. Materials and Methods

Synthesis of Si@SnO_2_: A total of 4 g of nano-Si (100 nm) and 6 g of SnCl_2_ (99.99%, AR) were mixed in a blender for 3 h. Then, the mixture was heated to 300 °C at a rate of 5 °C/min in a tubular furnace with an argon flow rate of 150 sccm and held for 2 h. Next, the sample was placed in a muffle furnace and heated to 300 °C at a rate of 5 °C/min and held for 2 h, resulting in a Si/SnO_2_ composite. Finally, 10 g of Si/SnO_2_ composite was added to 1000 mL of deionized water and stirred for 3 h with a magnetic stirrer. The uniformly stirred solution was then spray-dried (with the inlet temperature set to 180 °C and the outlet temperature set to 70 °C), yielding a spherical Si/tin oxide composite (Si@SnO_2_).

Preparation of Si@Sn@C composite: A small beaker was used to take 30 mL of toluene solution; then, 400 mg of pitch was added to the beaker, mixed with toluene, and stirred for 1 h on a magnetic stirrer to fully dissolve the pitch in the toluene solution. A total of 2 g of the prepared Si@SnO_2_ was added to the beaker and stirred for another 2 h to mix it evenly with the pitch. The mixture was then dried in an oil bath at 160 °C. The sample was then transferred to a tubular furnace with argon gas, heated to 400 °C at a rate of 5 °C/min, and held for 2 h, and 5 sccm of hydrogen gas was passed through, with a hydrogen-to-argon ratio of 1:30. After the holding period, the hydrogen gas was turned off, and the temperature was further increased to 950 °C and held for 2 h, resulting in a Si@Sn@C composite.

Preparation of Si@void@C: First, 6 M of HCl was prepared in a beaker; then, the Si@Sn@C was added to the beaker, left to stand for 24 h, and then filtered and washed. It was then dried in a blast drying oven at 60 °C to obtain the Si@void@C composite. Composites Si@void@C-1, Si@void@C-2, and Si@void@C-3 were synthesized by adjusting the weight ratio of Si to SnCl_2_ to 2:1, 2:3, and 2:5, respectively.

Characterization: The morphology, structure, and properties of the samples were characterized using the following methods: SEM (S-4800) and TEM (JEOL2100) were used to observe the morphology and structure of the samples. X-ray Diffraction (D8 Advance DaVinci) was used to collect the XRD spectra of the samples. Micro-Raman Spectroscopy (inVia-reflex) was used to collect the Raman spectra of the samples. XPS was conducted on a photoelectron spectrometer (AXIS ULTRA DLD, Shimadzu). TGA was carried out on a TGA209F1 (NETZSCH) at temperatures ranging from 25 °C to 900 °C with a heating rate of 10 °C/min in air.

Electrochemical characterization: The active material, carboxymethyl cellulose (CMC), and carbon black were mixed in a mass ratio of 8:1:1 to form a slurry, which was then coated on copper foil and dried in a vacuum oven at 80 °C for 12 h to prepare a working electrode for a half-cell. The loading mass of the active material was controlled at 1.4~1.6 mg cm^−2^. A lithium foil was used as the counter electrode, and polypropylene (PP) was used as the separator. The electrolyte used in the battery was 1 M LiPF_6_ (with a volume ratio of ethylene carbonate (EC), diethyl carbonate (DEC), and ethyl methyl carbonate (EMC) of 1:1:1). Using these components, a CR 2032 coin-type battery was assembled in a glove box filled with argon. Constant-current charge–discharge tests were conducted on a LAND battery test system, with a voltage range of 0.005–2.0 V. CV tests were recorded on a Solartron Metrology electrochemical workstation.

## 4. Conclusions

In summary, a yolk-shell-structured Si@void@C composite was prepared using a simple annealing process that involved coating nano- Si particles with Sn as a template. The composite was then spray-dried to form a spherical structure. Subsequently, pitch was uniformly coated on the surface through a liquid-phase method and carbonized in a high-temperature tube furnace. Finally, HCl was used to etch Sn, resulting in a yolk-shell-structured Si@void@C composite. The prepared yolk-shell structure provides reserved space for the volume expansion of Si, thereby alleviating volume expansion. The external carbon layer acts as a mechanical support to prevent Si from fracturing and prevents direct contact between Si and the electrolyte, further improving the overall conductivity of the composite. This results in Si@void@C having an excellent cycle stability and rate performance. After 100 cycles at 1.0 A g^−1^, the Si@void@C electrode retained a discharge capacity of 735.3 mAh g^−1^, which was 312.7 mAh g^−1^ higher than that of the Si@Sn@C electrode, demonstrating the superiority of the yolk-shell structure. Even at a high current density of 2.0 A g^−1^, Si@void@C still maintained a discharge capacity of 1238.5 mAh g^−1^. Hence, the use of Sn as a template facilitates the simple preparation of a yolk-shell-structured Si -carbon composite with superior electrochemical properties. This study presents the successful preparation of a Si@void@C composite with a high electrochemical performance, which was achieved through a simple and safe method. This achievement provides a valuable reference for the development of high-energy-density LIBs.

## Data Availability

Data are contained within the article and Supplementary Materials.

## Supplementary Materials

The following supporting information can be downloaded at: https://www.mdpi.com/article/10.3390/molecules29061301/s1, Figure S1: (a,b) SEM of Si nanoparticles; Figure S2: (a–c) SEM of Si@SnO2; Figure S3. (a,b) HRTEM of Si@void@C; Figure S4: (a–d) Element mapping images (Si, Sn and C) of Si@Sn@C; Figure S5: TG curve of pitch; Figure S6: XPS fine spectrum of Si@Sn@C: Sn 3d; Figure S7: CV profile of Si@Sn@C at 0.1 mV s^−1^; Figure S8. Enlarged Coulombic efficiency; Figure S9: Charge/discharge profiles of Si@Sn@C between 0.1 and 2 A g^−1^; Table S1. The performance of different silicon-based anodes; Table S2. Abbreviations of the vocabulary. References [51,52,53,54,55,56,57,58,59,60] are cited in the Supplementary Materials.
