# Supplementary material for: Simple and Safe Synthesis of Yolk-Shell-Structured Silicon/Carbon Composites with Enhanced Electrochemical Properties
Source: Molecules. 2024 Mar 14;29(6):1301. doi: 10.3390/molecules29061301 (PMC10976018; doi:10.3390/molecules29061301)
Supplement: Supplementary file 1 [file molecules-29-01301-s001.zip › molecules-2913636-supplementary.pdf]

molecules

Supplementary Materials

Simple and Safe Synthesis of Yolk-Shell-Structured Silicon/Carbon Composites with Enhanced Electrochemical Properties

Jinhuan Li [1,2], Min Wu [1], Quan Du [1], Gangpeng Zhai [1] and Haiyong He [1,*]

[1] Ningbo Institute of Materials Technology and Engineering, Chinese Academy of Sciences, Ningbo 315201, China
[2] University of Chinese Academy of Sciences, Beijing 101400, China
* Correspondence: hehaiyong@nimte.ac.cn

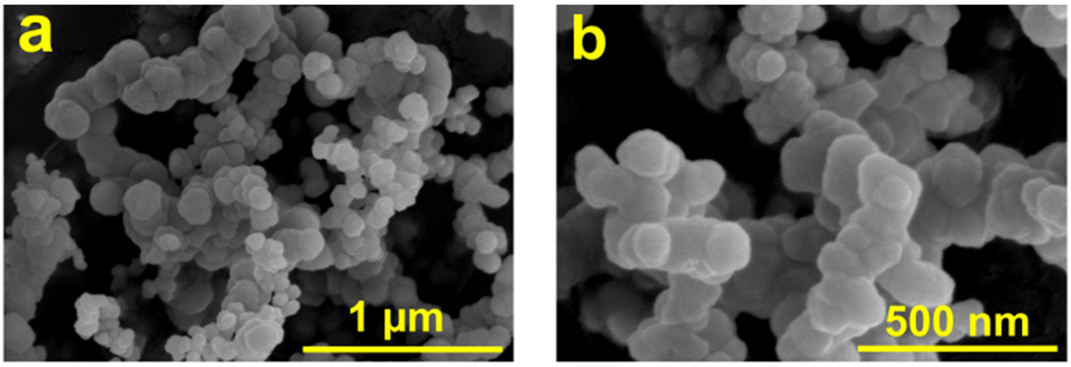

Figure S1. (**a**,**b**) SEM of Si nanoparticles.

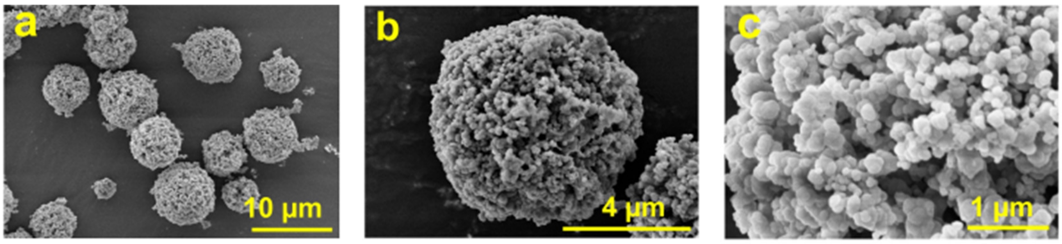

Figure S2. (**a**–**c**) SEM of Si@SnO_2.

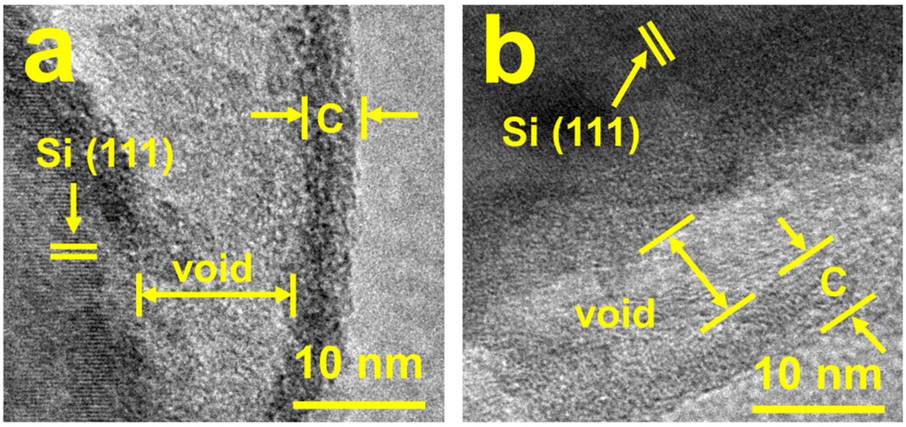

Figure S3. (**a**,**b**) HRTEM of Si@void@C.

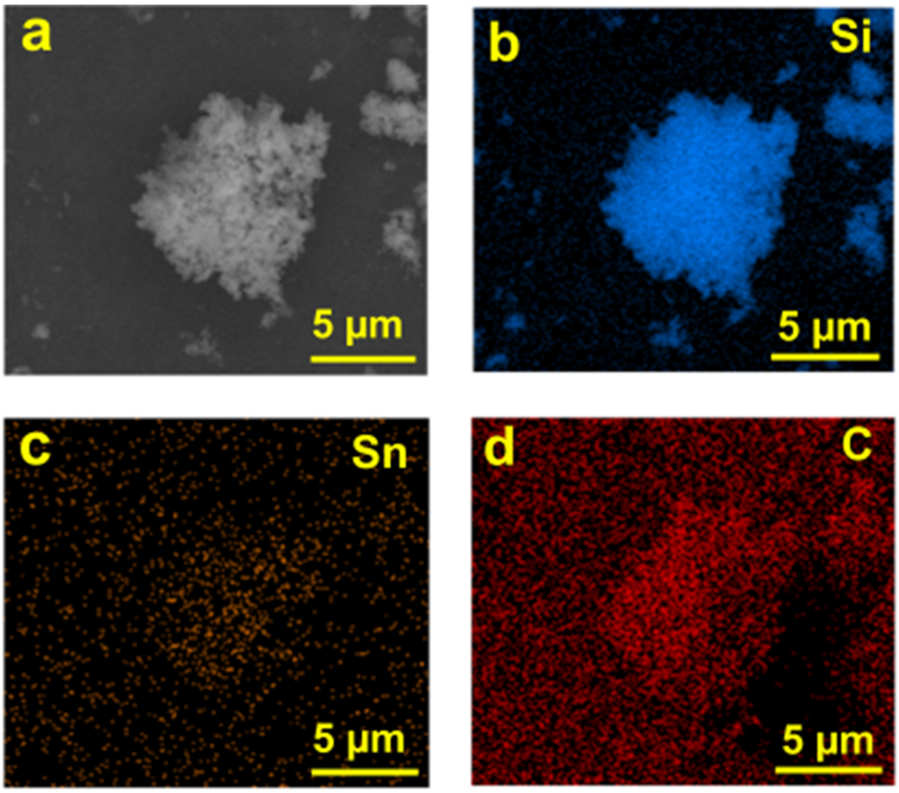

Figure S4. (**a**–**d**) Element mapping images (Si, Sn and C) of Si@Sn@C.

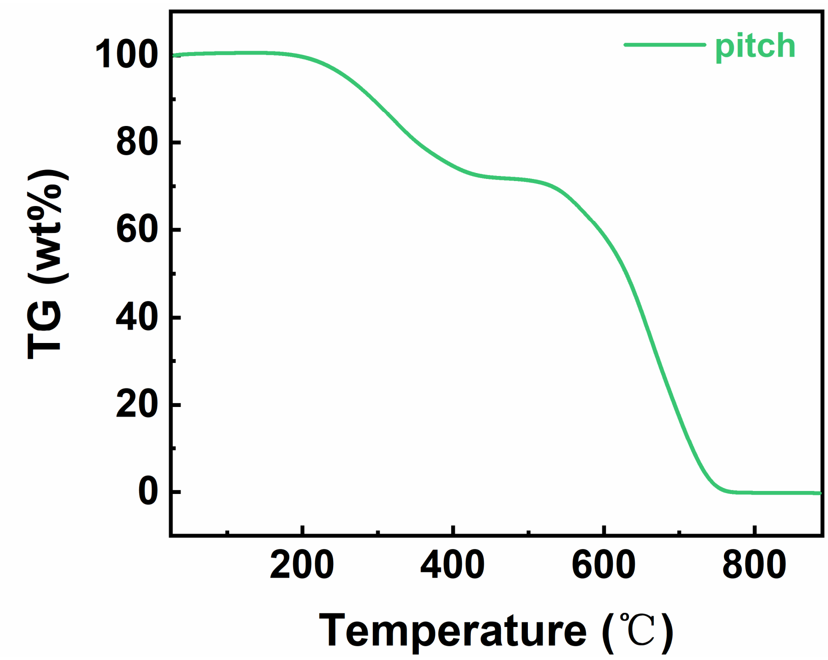

Figure S5. TG curve of pitch.

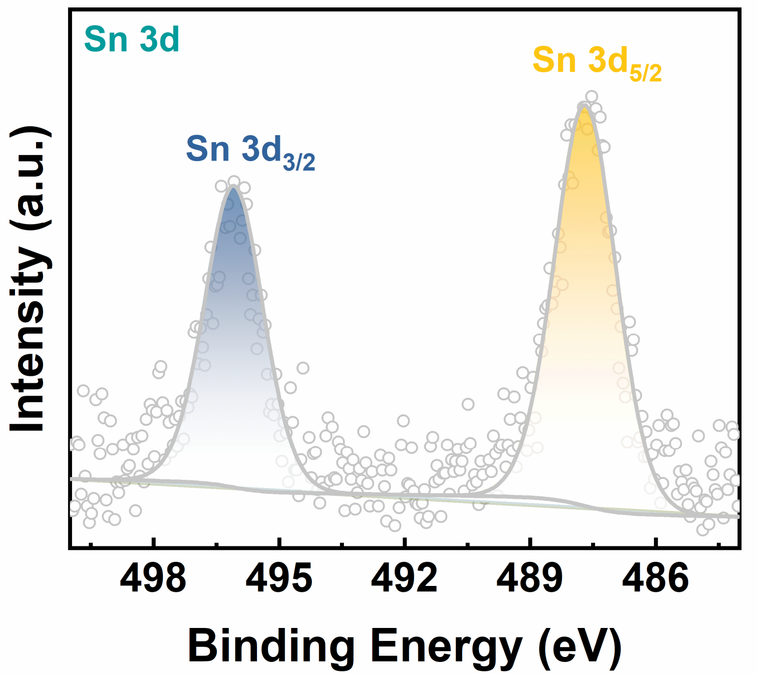

Figure S6. XPS fine spectrum of Si@Sn@C: Sn 3d.

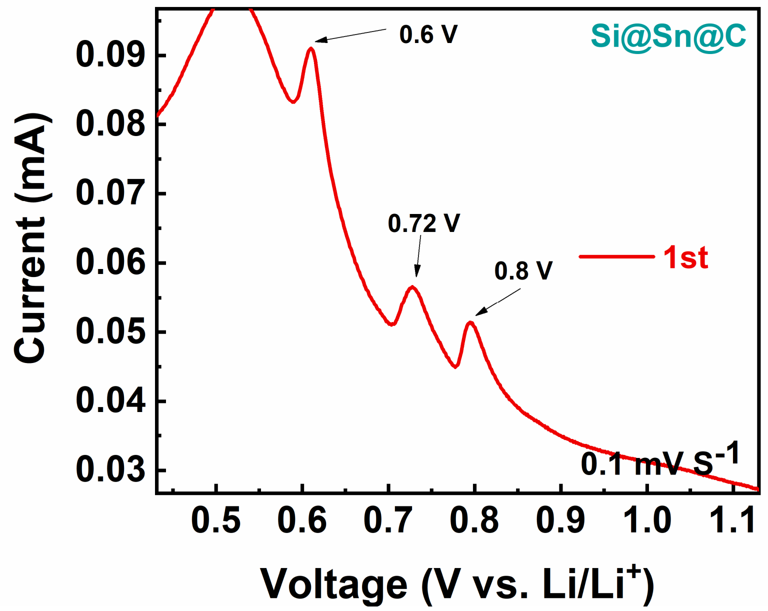

Figure S7. CV profile of Si@Sn@C at 0.1 mV s^{-1}.

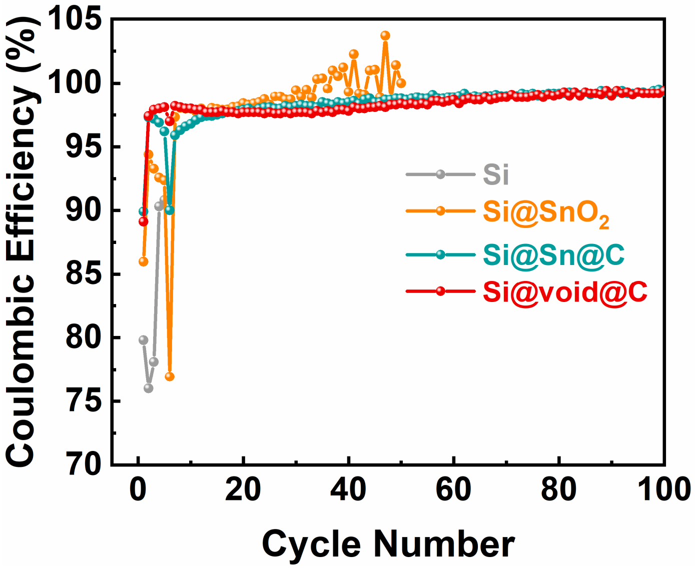

Figure S8. Enlarge Coulombic Efficiency.

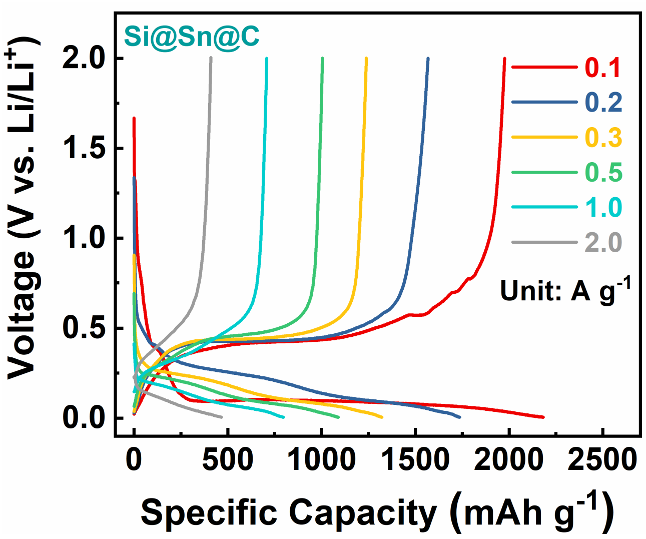

Figure S9. Charge/discharge profiles of Si@Sn@C at current densities between 0.1 and 2 A $g^{-1.}$

Table S1. The performance of different silicon-based anodes.

Samples	1st Discharge capacity (mAh g^{-1})	1st Columbic efficiency (%)	Discharge Capacity (mAh g^{-1})	Cycle number	Ref.
Si/CNTs@FG	1204	62.3	970	100	[1]
Si/Cu-Zn(ox)/Cu	/	50	800	100	[2]
Si/C nanosphere	1421	66	738	120	[3]
Our work	2442	89	735	100	
Core-shell Si@N	2602	75.4	725	100	[4]
HSi@C	1610	70	700	100	[5]
Porous Si@C	/	76.4	680	50	[6]
SiO_x/C	887	57.19	550	180	[7]
CNTs-carbon coated SiO_x	1741	80.7	458	100	[8]
SiO_x/G/SnO_2	/	62.2	424.6	110	[9]
layer LSM	579	34.5	272.3	20	[10]

Table S2. Abbreviations for the vocabulary.

1.	**vocabulary**	2.	**abbreviations**
3.	silicon	4.	Si
5.	lithium-ion batteries	6.	LIBs
7.	tin oxide	8.	SnO_2
9.	concentrated hydrochloric acid	10.	HCl
11.	hydrofluoric acid	12.	HF
13.	Silicon@void@Carbon	14.	Si@void@C
15.	tin chloride	16.	$SnCl_2$
17.	Scanning Electron Microscopy	18.	SEM
19.	Transmission Electron Microscopy	20.	TEM
21.	X-ray diffraction	22.	XRD
23.	Thermogravimetric analysis	24.	TGA
25.	X-ray photoelectron spectroscopy	26.	XPS
27.	cyclic voltammetry	28.	CV
29.	electrochemical impedance spectra	30.	EIS
31.	initial Coulombic Efficiencies	32.	ICE
33.	silicon/tin oxide composite	34.	Si@SnO_2
35.	carboxymethyl cellulose	36.	CMC
37.	polypropylene	38.	PP
39.	ethylene carbonate	40.	EC
41.	diethyl carbonate	42.	DEC
43.	ethyl methyl carbonate	44.	EMC

References

45. Wang, X.; Wen, K.; Chen, T.; Chen, S.; Zhang, S. Supercritical Fluid-Assisted Preparation of Si/CNTs@FG Composites with Hierarchical Conductive Networks as a High-Performance Anode Material. *Applied Surface Science* **2020**, 522, 146507, doi:10.1016/j.apsusc.2020.146507.
46. He, Y.; Ye, Z.; Chamas, M.; Sougrati, M.T.; Lippens, P.-E. Si/Cu-Zn(Ox)/C Composite as Anode Material for Li-Ion Batteries. *Solid State Ionics* **2021**, 372, 115774, doi:10.1016/j.ssi.2021.115774.
47. Li, Z.; Li, Z.; Zhong, W.; Li, C.; Li, L.; Zhang, H. Facile Synthesis of Ultrasmall Si Particles Embedded in Carbon Framework Using Si-Carbon Integration Strategy with Superior Lithium Ion Storage Performance. *Chemical Engineering J.* **2017**, 319, 1–8, doi: 10.1016/j.cej.2017.02.141.
48. Shen, X.; Jiang, W.; Sun, H.; Wang, Y.; Dong, A.; Hu, J.; Yang, D. Ionic Liquid Assist to Prepare Si@N-Doped Carbon Nanoparticles and Its High Performance in Lithium Ion Batteries. *J. Alloys and Compounds* **2017**, 691, 178–184, doi:10.1016/j.jallcom.2016.08.265.
49. Ashuri, M.; He, Q.; Liu, Y.; Zhang, K.; Emani, S.; Sawicki, M.S.; Shamie, J.S.; Shaw, L.L. Hollow Silicon Nanospheres Encapsulated with a Thin Carbon Shell: An Electrochemical Study. *Electrochimica Acta* **2016**, 215, 126–141, doi:10.1016/j.electacta.2016.08.059.
50. Dong, H.; Fu, X.; Wang, J.; Wang, P.; Ding, H.; Song, R.; Wang, S.; Li, R.; Li, S. In-Situ Construction of Porous Si@C Composites with LiCl Template to Provide Silicon Anode Expansion Buffer. *Carbon* **2021**, 173, 687–695, doi:10.1016/j.carbon.2020.11.042.
51. Zhang, B.; Wang, H.; Liu, C.; Li, D.; Kim, H.-K.; Harris, C.; Lao, C.; Abdelkader, A.; Xi, K. Facile Mechanochemical Synthesis of Non-Stoichiometric Silica-Carbon Composite for Enhanced Lithium Storage Properties. *J. Alloys and Compounds* **2019**, 801, 658–665, doi:10.1016/j.jallcom.2019.06.101.
52. Li, G.; Huang, L.-B.; Yan, M.-Y.; Li, J.-Y.; Jiang, K.-C.; Yin, Y.-X.; Xin, S.; Xu, Q.; Guo, Y.-G. An Integral Interface with Dynamically Stable Evolution on Micron-Sized SiOx Particle Anode. *Nano Energy* **2020**, 74, 104890, doi:10.1016/j.nanoen.2020.104890.
53. Yuan, T.; Tang, R.; Xiao, F.; Zuo, S.; Wang, Y.; Liu, J. Modifying SiO as a Ternary Composite Anode Material((SiOx/G/SnO2)@C) for Lithium Battery with High Li-Ion Diffusion and Lower Volume Expansion. *Electrochimica Acta* **2023**, 439, 141655, doi:10.1016/j.electacta.2022.141655.
54. 10. Gao, R.; Tang, J.; Terabe, K.; Yu, X.; Sasaki, T.; Hashimoto, A.; Asano, K.; Suzuki, M.; Nakura, K. Preparation of Layered Si Materials as Anode for Lithium-Ion Batteries. *Chemical Physics Letters* **2019**, 730, 198–205, doi:10.1016/j.cplett.2019.06.010.
